# Supplementary material for: LMO7 deficiency reveals the significance of the cuticular plate for hearing function
Source: Nat Commun. 2019 Mar 8;10:1117. doi: 10.1038/s41467-019-09074-4 (PMC6408450; doi:10.1038/s41467-019-09074-4)
Supplement: Supplementary file 1 — Supplementary Information [file 41467_2019_9074_MOESM1_ESM.pdf]

Supplementary Information

Du et al.,

LMO7 deficiency reveals the significance of the cuticular plate for hearing function

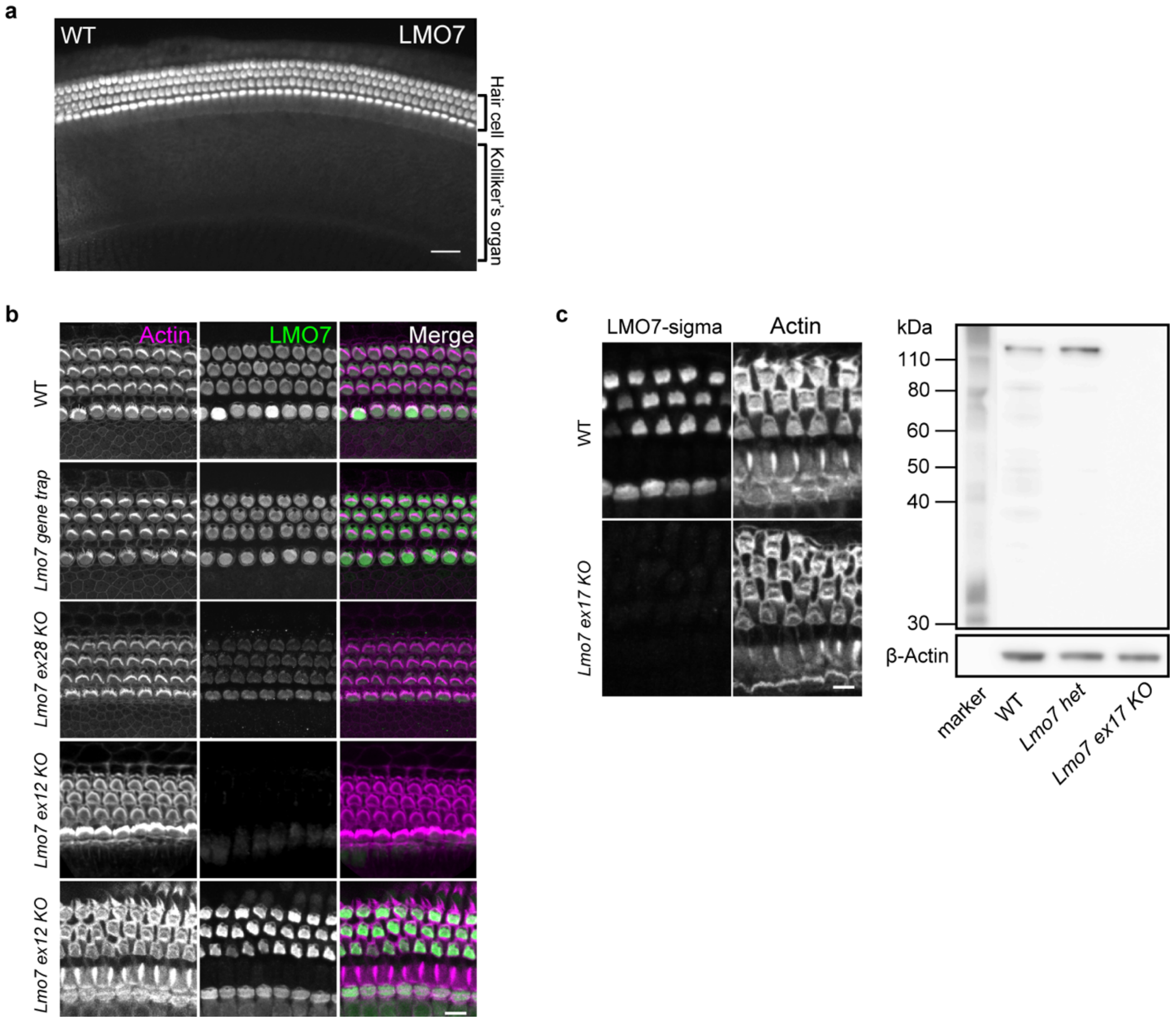

Supplementary Figure 1. a, Immunohistochemical localization of LMO7 in P4 WT organ of Corti demonstrates specific expression in hair cells. Scale bar, 20 µm. **b.** LMO7 expression pattern in different mutant mice at P4: WT, *Lmo7 gene trap*, *Lmo7 ex28 KO*, *Lmo7 ex12 KO* and P24 *Lmo7 ex12 KO* mice cochlea. **C.** Immunohistochemistry and western blot (Lung) using the Sigma anti-LMO7 antibody, demonstrating lack of immunoreactivity in the *Lmo7 exon17 KO* tissue. The epitope recognized by this antibody is indicated in Fig. 3a. Scale bar, 5 µm.

a

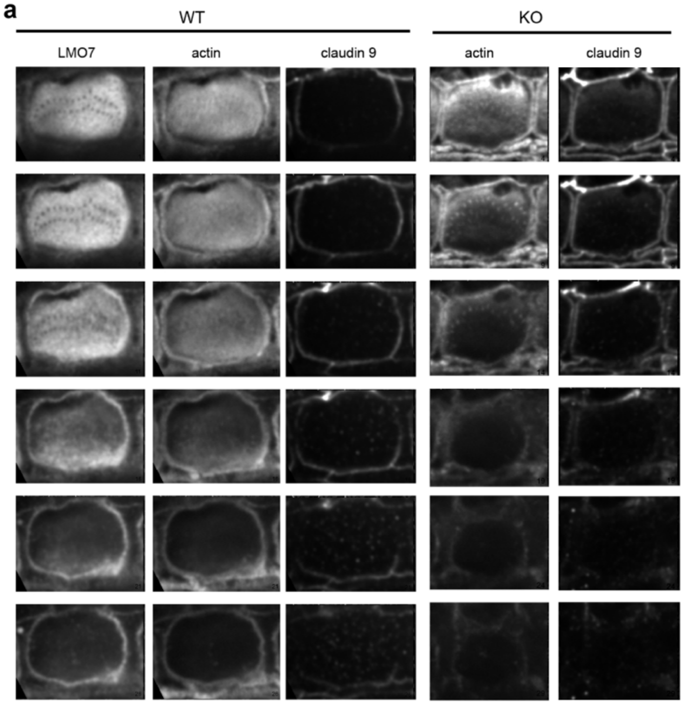

b

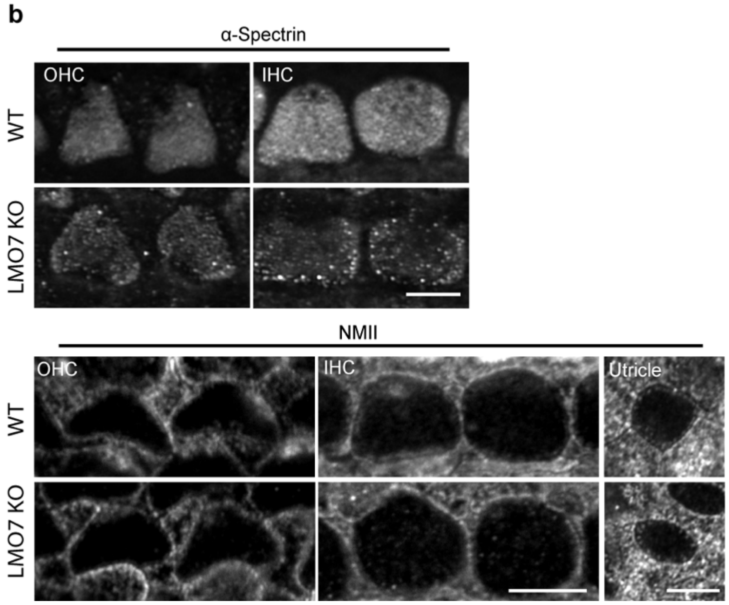

Supplementary Figure 2. a. Immunoreactivity of LMO7, actin and the tight junction protein claudin 9 at different depth levels in the cuticular plate. Both actin and claudin 9 signals extend less deeply in the *Lmo7 exon17 KO* as compared to WT counterparts. This is an extended version of Fig. 4e.
b. Immunocytochemical analysis of spectrin and NMII in cochlea and utricle of WT and *Lmo7 exon17 KO* at P6.

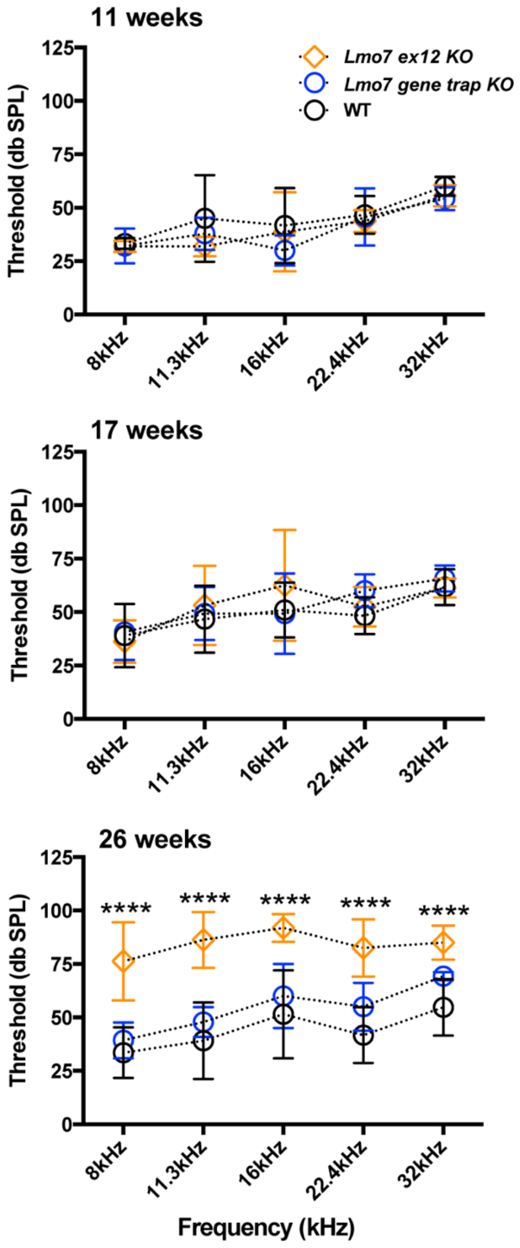

Supplementary Figure 3. Auditory brainstem response analysis demonstrates that *Lmo7 exon12 KO* but not *Lmo7 gene trap* mice develop late onset, progressive hearing loss. At 11 weeks: N=6 for WT, N=7 for *Lmo7 gene trap*, N=8 for *Lmo7 exon12 KO*. At 17 weeks: N=15 for WT, N=7 for *Lmo7 gene trap*, N=8 for *Lmo7 exon12 KO*. At 26 weeks: N=17 for WT, N=7 for *Lmo7 gene trap*, N=8 for *Lmo7 exon12 KO*. Both male and female mice were used. Error bars indicate SD, ****p value<0.0001 according to ANOVA test, followed by Tukey post-hoc analysis
