# Supplementary material for: LMO7 deficiency reveals the significance of the cuticular plate for hearing function
Source: Nat Commun. 2019 Mar 8;10:1117. doi: 10.1038/s41467-019-09074-4 (PMC6408450; doi:10.1038/s41467-019-09074-4)
Supplement: Supplementary file 3 — Description of Additional Supplementary Information [file 41467_2019_9074_MOESM3_ESM.pdf]

Description of Additional Supplementary Files

File Name: Supplementary Data 1
Description: Full list of co-immunoprecipitated proteins from WT organ of Corti.

File Name: Supplementary Data 2
Description: Full list of co-immunoprecipitated proteins from *Lmo7 KO* organ of Corti.
