# Supplementary material for: LMO7 deficiency reveals the significance of the cuticular plate for hearing function
Source: Nat Commun. 2019 Mar 8;10:1117. doi: 10.1038/s41467-019-09074-4 (PMC6408450; doi:10.1038/s41467-019-09074-4)
Supplement: Supplementary file 6 — Reporting summary [file 41467_2019_9074_MOESM6_ESM.pdf]

nature research

Corresponding author(s): Jung-Bum Shin

Last updated by author(s): Feb 6, 2019

Reporting Summary

Nature Research wishes to improve the reproducibility of the work that we publish. This form provides structure for consistency and transparency in reporting. For further information on Nature Research policies, see Authors & Referees and the Editorial Policy Checklist.

Statistics

For all statistical analyses, confirm that the following items are present in the figure legend, table legend, main text, or Methods section.

n/a	Confirmed	
☐	☒	The exact sample size (*n*) for each experimental group/condition, given as a discrete number and unit of measurement
☐	☒	A statement on whether measurements were taken from distinct samples or whether the same sample was measured repeatedly
☐	☒	The statistical test(s) used AND whether they are one- or two-sided<br>*Only common tests should be described solely by name; describe more complex techniques in the Methods section.*
☐	☒	A description of all covariates tested
☒	☐	A description of any assumptions or corrections, such as tests of normality and adjustment for multiple comparisons
☐	☒	A full description of the statistical parameters including central tendency (e.g. means) or other basic estimates (e.g. regression coefficient) AND variation (e.g. standard deviation) or associated estimates of uncertainty (e.g. confidence intervals)
☐	☒	For null hypothesis testing, the test statistic (e.g. *F*, *t*, *r*) with confidence intervals, effect sizes, degrees of freedom and *P* value noted<br>*Give P values as exact values whenever suitable.*
☒	☐	For Bayesian analysis, information on the choice of priors and Markov chain Monte Carlo settings
☒	☐	For hierarchical and complex designs, identification of the appropriate level for tests and full reporting of outcomes
☒	☐	Estimates of effect sizes (e.g. Cohen's *d*, Pearson's *r*), indicating how they were calculated

Our web collection on statistics for biologists contains articles on many of the points above.

Software and code

Policy information about availability of computer code

Data collection	Image data collection was performed using the Zen software (Zeiss) or managed through NIS-Elements software (Nikon Instruments). For ABRs, the SmartEP and for DPOAEs, the SmartOAE software were used to collect data (Intelligent Hearing Systems).
Data analysis	Scaffold viewer version 4.8.8 (free download) was used visualize and analyze Co-IP/MS data.<br>Analysis of confocal images were performed using ImageJ or the NIS-Elements software (Nikon).<br>Graph Pad Prism V7 for statistical analysis.

For manuscripts utilizing custom algorithms or software that are central to the research but not yet described in published literature, software must be made available to editors/reviewers. We strongly encourage code deposition in a community repository (e.g. GitHub). See the Nature Research guidelines for submitting code & software for further information.

Data

Policy information about availability of data

All manuscripts must include a data availability statement. This statement should provide the following information, where applicable:

- Accession codes, unique identifiers, or web links for publicly available datasets
- A list of figures that have associated raw data
- A description of any restrictions on data availability

Table 1 in the manuscript is a summary of the data resulting from the co-immunoprecipitation/mass spectrometry experiment. The raw data (excel files) are available in the Supplemental data. All other data described in the manuscript are available upon request.

Field-specific reporting

Please select the one below that is the best fit for your research. If you are not sure, read the appropriate sections before making your selection.

☒ Life sciences ☐ Behavioural & social sciences ☐ Ecological, evolutionary & environmental sciences

For a reference copy of the document with all sections, see nature.com/documents/nr-reporting-summary-flat.pdf

Life sciences study design

All studies must disclose on these points even when the disclosure is negative.

Sample size	Sample size for the hearing tests were determined using power analysis. Historical variance of ABR thresholds in the Shin lab was used for the power analysis, with an expectation of a P-value of 0.05. In case no historical data was available, pilot experiments were performed to estimate variance (e.g. for gentamicin uptake assay in Fig.7).
Data exclusions	no data were excluded
Replication	All findings presented in the present study either underwent rigorous statistical treatment (involving multiple independent replications) or in case of qualitative statements (e.g. differences reported in light and electron microscopy studies), findings were replicated multiple times, by multiple experimenters, and for the COS-7 expression studies, in two different laboratories (Shin and Kachar labs).
Randomization	Randomization for mouse work was achieved by distributing experimental groups (Lmo7 ko or WT) across cages (in other words, cages did not indicate experimental group). For practical reasons, such randomization was not performed for sexes: mice are housed separated by sex, so when performing hearing tests, the experimenter was aware of the sex of the tested mouse.
Blinding	Data was collected and analyzed in a blinded fashion for most quantifications described in the manuscript except the functional hearing tests. For the longitudinal hearing tests that were performed over a course of 6 months, the experimenter often inadvertently "memorized" tag numbers and their corresponding genotypes, despite initial blinding, because data was analyzed at each age the hearing performance was assessed. To prevent bias, however, analysis of the data (threshold determination based on recorded traces) was confirmed by an independent experimenter.

Reporting for specific materials, systems and methods

We require information from authors about some types of materials, experimental systems and methods used in many studies. Here, indicate whether each material, system or method listed is relevant to your study. If you are not sure if a list item applies to your research, read the appropriate section before selecting a response.

Materials & experimental systems

n/a	Involved in the study	
☐	☒	Antibodies
☐	☒	Eukaryotic cell lines
☒	☐	Palaeontology
☐	☒	Animals and other organisms
☒	☐	Human research participants
☒	☐	Clinical data

Methods

n/a	Involved in the study	
☒	☐	ChIP-seq
☒	☐	Flow cytometry
☒	☐	MRI-based neuroimaging

Antibodies

Antibodies used	rabbit anti-LMO7 (M-300, sc-98422; Lot unknown, discontinued, Santa Cruz Biotechnology), mouse anti-LMO7 (B-7, sc-376807, lot# A1315, Santa Cruz Biotechnology, replacement product of rabbit M-300 antibody), rabbit anti-LMO7 (Sigma Prestige antibody, lot# R09596, HPA020923). M-300 and B-7 were raised against the same epitope and in our immunohistochemistry experiments, yielded highly comparable results. Mouse anti-Spectrin alpha chain (MAB1622; lot# 2586521, MilliporeSigma), V5 Tag Antibody (R960-25, lot# 1900119, Invitrogen), rabbit anti-TRIOBP (16124-1-AP, lot# 7387, Proteintech Group), rabbit anti-MYO7A (111501, lot# 111501, Proteus BioSciences), mouse anti-Gentamicin (16102, lot# 111092-060603, QED Bioscience Inc.), rabbit anti-NMII (PRB-444P-100, lot# D04, Covance), anti-Claudin-9 (Custom-made rabbit polyclonal antibody PB209, PRGPRLGYSIPSRSGA). The Claudin-9 antibody was originally described and validated in Nunes et al. reference 56 in manuscript
Validation	The following antibodies were validated by confirming absence of immunoreactivity in KO mice or samples: rabbit anti-LMO7 (M-300), rabbit anti-LMO7 (Sigma), mouse anti-LMO7 (B-7), rabbit anti-MYO7A . The custom-made Claudin-9 antibody was originally described and validated in Nunes et al. (reference 56 in manuscript). The rabbit TRIOBP antibody produces immunoreactivities in the inner ear consistent with previous reports of TRIOBP localization and expression patterns (Kitajiri ... Friedman. 2010, Cell). Same applies to the rabbit NMII antibody (Francis..Shin, 2013, Journal of Neuroscience). All other antibodies are commercially antibodies with extensive validation data on the product's website.

Eukaryotic cell lines

Policy information about cell lines

Cell line source(s)	COS-7 cell line
Authentication	COS-7 expression experiments were performed in the Shin and Kachar labs, using independent batches. Results were highly consistent.
Mycoplasma contamination	not tested
Commonly misidentified lines (See ICLAC register)	not applicable

Animals and other organisms

Policy information about studies involving animals; ARRIVE guidelines recommended for reporting animal research

Laboratory animals	This study involved the use of the following strains of mice:<br>- Lmo7 exon17 KO, on C57BL6/J or CBA/J backgrounds<br>- Lmo7 exon12 (4 generations backcrossed into C57BL6/J<br>- Lmo7 exon28 (4 generations backcrossed into C57BL6/J<br>- Lmo7 GFP11 KI (4 generations backcrossed into C57BL6/J<br>- Lmo7 gene trap mice were reconstituted by IVF using frozen sperm obtained from Texas A&M (mouse ID: Lmo7Gt(IST10208D3)Tigm), on C57BL6/J background<br>Much of Fig.3 and supplementary figure 1 is devoted to the validation of these mice.<br>- Tmc1/2 DOKO mice were provided by Dr. Andrew Griffith, on C57BL6/J background (Kawashima ... Griffith, 2011, JCI)
Wild animals	we did not use wild animals
Field-collected samples	we did not collect field samples
Ethics oversight	The protocol for care and use of animals was approved by the University of Virginia Animal Care and Use Committee. The University of Virginia is accredited by the American Association for the Accreditation of Laboratory Animal Care

Note that full information on the approval of the study protocol must also be provided in the manuscript.
